# Molecular Mimicry by the Tick-Borne Encephalitis Virus E Protein: A Hidden Link to Autoimmunity

**DOI:** 10.3390/ijms27114745

**Published:** 2026-05-25

**Authors:** Anna M. Timofeeva, Ksenia S. Aulova, Yana S. Ulyanova, Mark M. Melamud, Sergey G. Arkhipov, Elena I. Krasnova, Georgy A. Nevinsky

**Affiliations:** 1 SB RAS Institute of Chemical Biology and Fundamental Medicine, 630090 Novosibirsk, Russia; 2 Department of Infectious Diseases, Novosibirsk State Medical University, 630091 Novosibirsk, Russia; 3 SRF “SKIF”, 630559 Koltsovo, Russia

**Keywords:** tick-borne encephalitis, molecular mimicry, antibodies, autoimmunity, E protein, HLA, autoantibodies, B-cell epitopes, T-helper epitopes, cross-reactivity, bioinformatic analysis, vaccination, Tick-E-Vac

## Abstract

In this study, we combined computational predictions with experimental validation as a hybrid strategy to explore whether the E protein of tick-borne encephalitis virus (TBEV) possesses autoimmune potential. Using in silico homology searches, we identified two viral epitopes (evglekl and vtgtqgt) within the TBEV E protein that share sequence identity with fragments of the human proteins DNAH7 and CSMD2. Antibodies against these epitopes were detected in the plasma of a subset of patients after natural TBEV infection. Notably, no such antibodies were found in recipients of the Tick-E-Vac vaccine, indicating that the current vaccine does not induce cross-reactive humoral responses to these epitopes. Further computational analysis predicted that these epitopes could be presented by HLA class II molecules (alleles DRB1*09:01 and DRB1*07:01), which are known to be associated with autoimmune pathologies. Molecular dynamics simulations confirmed stable binding of the peptides within the HLA grooves, with favorable binding energies. These findings suggest a possible involvement of T-helper cells in the autoreactive process. Natural TBEV infection can give rise to antibodies against epitopes homologous to human proteins, particularly in genetically predisposed hosts. While such homology alone does not predict the onset of autoimmune disease, it represents a risk factor.

## 1. Introduction

The causative agent of tick-borne encephalitis (TBE) is an RNA virus belonging to the *Flaviviridae* family. The tick-borne encephalitis virus (TBEV) is transmitted by ticks of the genus *Ixodes* [1]. Based on the envelope gene sequence, the virus is taxonomically classified into Siberian (Sib-TBEV), Far Eastern (FE-TBEV), and European (EU-TBEV) subtypes, with two additional subtypes, Baikalian and Himalayan, recently reported [2,3]. Encephalitis caused by European subtype viruses typically follows a mild course, with a case fatality rate of 1–5%, whereas Far Eastern strains cause severe encephalitis with a case fatality rate of 20–60%. Siberian subtype isolates cause less severe disease but have a tendency to lead to chronic infections [4,5]. Meanwhile, in Russia, the number of medical consultations for tick bites has been steadily increasing and exceeded half a million cases in 2022 [6,7].

There are currently no specific treatments for TBE. Recent studies indicate an expansion of geographical zones, the emergence of new foci, and a shift in the range of *Ixodes* ticks, which may increase the risk of TBEV infection [8,9,10]. Vaccination is considered the most reliable protection against this infection: effective and accessible TBE vaccines for adults and children have been developed. However, vaccination coverage in European countries and Russia remains insufficient [11].

Among environmental factors, viruses are considered the primary agents capable of triggering autoimmune processes in individuals with a genetic predisposition [12]. Theoretically, the immune system should not produce antibodies against self-proteins. To evade the immune response, viruses mutate so that some fragments of their proteins exhibit similarity to human proteins. This phenomenon is termed molecular mimicry [13,14,15]. Numerous examples documented in the literature confirm the association between viral infections and autoimmune diseases, such as Epstein–Barr virus and multiple sclerosis [16,17,18,19], and Coxsackievirus and type I diabetes [20,21]. During the COVID-19 pandemic, many cases of autoimmune diseases associated with COVID-19 were reported [22,23,24,25], and similarity between some SARS-CoV-2 protein sequences and human proteins was demonstrated [26,27,28,29].

The literature describes the development of autoimmune diseases following infection with various flaviviruses. For example, the outbreak of Zika virus infections in Polynesia in 2013 was accompanied by a surge in acute inflammatory neuropathy, Guillain–Barré syndrome [30]. Another study found higher titers of antibodies against the Zika virus in patients who developed Guillain–Barré syndrome compared to patients with uncomplicated infection [31]. Another study showed that patients infected with the Zika virus who subsequently developed Guillain–Barré syndrome had higher levels of anti-ganglioside autoantibodies compared to patients with an uncomplicated course of the disease [32].

A number of studies have shown an association between Dengue fever and an increased risk of developing certain autoimmune diseases, such as multiple sclerosis, myasthenia gravis, autoimmune encephalomyelitis, systemic lupus erythematosus, post-infectious arthritis, systemic vasculitis, and autoimmune myositis [33]. In cases of severe Dengue fever, autoantibodies produced in response to the virus can cross-react with autoantigens on endothelial cells, platelets, or molecules involved in blood coagulation [34,35]. Chikungunya, West Nile, and Japanese encephalitis viruses have also been associated with the development of autoimmune encephalomyelitis [36,37,38].

The E protein, a surface glycosylated structural protein of flaviviruses, is a target for neutralizing antibodies [39] and is responsible for viral fusion with the host cell plasma membrane, as well as for the assembly and stability of viral particles [40,41]. The similarity between the Zika virus E protein and the human complement component C1q has been shown in silico, and antibodies against C1q have been detected in the blood plasma of patients infected with the virus [42]. It should be noted that the E protein exhibits approximately 40% amino acid identity between related flaviviruses [43].

Several cases of autoantibodies against the N-methyl-D-aspartate receptor (NMDAR) have been reported in patients with Japanese encephalitis [44,45] and TBE [46]. Two case reports have described how TBE caused the development of anti-NMDAR encephalitis [47,48].

To date, the role of the TBEV in the development of autoimmunity has been studied very poorly. Given the growing body of evidence indicating a link between various flaviviral infections and autoimmune diseases, this work is devoted to the study of the autoimmune potential of the E protein of the envelope of the TBEV. The autoimmune potential of the TBE virus E protein was assessed here using immunoinformatics approaches. The presence of antibodies to the identified potential epitopes was also investigated in blood samples from patients after infection and vaccinated individuals. This work describes the possibility of both the formation of cross-reactive antibodies and the activation of autoreactive T-cell responses through the mechanism of molecular mimicry.

## 2. Results

### 2.1. Predicting Regions of Molecular Mimicry Targeted by B Cells

For this analysis, three E protein sequences of the TBEV were selected from the GenBank database: two belonging to the Far-Eastern subtype and one to the Siberian subtype. These proteins exhibit a high degree of homology, with sequence identity ranging from 96.6% to 99.2%. Multiple sequence alignment revealed only single amino acid substitutions between them (Table 1).

The analysis of similarity between viral and human proteins was based on the concept of SCS. This approach involves splitting the primary protein sequence into short peptides, shifted relative to each other by one amino acid residue [15,49,50]. The choice of peptide length for epitope mapping is critical. Although antibodies theoretically recognize fragments as short as five amino acids [51,52], in practice, longer sequences are used to achieve an optimal balance between specificity and sensitivity [53,54,55]. Accordingly, a length of 7 amino acids was selected for this study.

Each of the three E proteins was dissected in silico into a set of 7-mers. A unified library of unique 7-mers was then created by removing duplicates, resulting in a library of 593 heptapeptides. Subsequently, each 7-mer in the library was searched for matches against the entire human proteome using the UniProtKB database. As a result, 9 proteins containing regions of identity with the E protein were identified (Table 2).

SCSs may be located either within epitopes or outside them. Only SCSs located within epitopes can be recognized by the immune system and lead to the development of an autoimmune response [56,57,58]. Therefore, the concurrent presence of SCSs within the epitopes of both the viral protein and human proteins was subsequently verified using the ABCpred Prediction Server [59,60]. As a result, 5 human proteins containing regions identical to the TBEV E protein within epitope structures were identified: DNAH7, STRA8, SETD2, ZIM2, and CSMD2.

ZIM2 and SETD2 are predominantly nuclear proteins, while STRA8 is cytoplasmic; therefore, their direct interaction with immune cells in the intact state is substantially limited. However, in autoimmune pathologies, a broad spectrum of antinuclear antibodies is produced (Sm, RNP, Ro/SSA, La/SSB, etc.), and it cannot be entirely excluded that during processes accompanying viral encephalitis (such as cell death, necrosis, or NETosis) these intracellular antigens are released and become accessible to the immune system. Nevertheless, in the present study, the proteins SETD2, ZIM2, and STRA8 were excluded from further analysis due to their predominantly intracellular localization.

The *DNAH7* gene encodes the axonemal dynein heavy chain 7 protein. This protein is a key component of the inner dynein arm within axonemes of respiratory cilia, which facilitates mucus clearance along the airways [61]. Expression levels of DNAH7 are markedly downregulated in human bronchial epithelial cells upon infection with SARS-CoV-2, MERS-CoV, and influenza A virus (H1N1) [62,63]. Moreover, mutations in DNAH7 have been linked to a higher risk of COVID-19 mortality [64].

CSMD2 is a membrane protein. The domain containing the region of identity with the E protein is located in the extracellular space. The *CSMD2* gene encodes a protein likely involved in the regulation of the complement cascade of the immune response [65,66]. Studies have shown that the development and maintenance of dendrites and dendritic spines in the brain depend on CSMD2 [67]. Furthermore, CSMD2 negatively correlated with MHC-I molecules and positively correlated with MHC-II molecules. CSMD2 expression also negatively correlated with the infiltration of antitumor immune cells, including macrophages and natural killer cells, and was positively associated with immune evasion [68].

Therefore, two viral epitopes (evglekl, vtgtqgt) may theoretically be involved in triggering a B-cell autoreactive response, resulting in the production of anti-DNAH7 and anti-CSMD2 antibodies. Their potential contribution to the development of autoreactive responses upon TBEV infection cannot be excluded.

### 2.2. Experimental Evaluation of Individual Epitope Immunodominance

Next, the immunodominance of four viral epitopes was experimentally evaluated in patient plasma samples. This evaluation enables the assessment of epitope recognition and the potential for an autoimmune response. Three groups of subjects were established for this study: patients diagnosed with tick-borne encephalitis (TBE), individuals vaccinated with the Tick-E-Vac vaccine (Vac), and healthy control donors (Con).

The TBE group consisted of 22 patients aged 21 to 69 years, who were hospitalized for TBE at the City Infectious Diseases Clinical Hospital No. 1 (Novosibirsk). The Vac group included 14 individuals aged 26 to 63 years who reported vaccination within the previous 3 years and had high antibody titers against TBEV. The Con group comprised 14 individuals aged 27 to 57 years who reported no history of vaccination and had negative antibody titers against TBEV.

In the present study, an immunodominance analysis of linear epitopes was employed. The peptide-based approach enables mapping of homologous linear epitopes with high precision, eliminating the masking effect of other regions of the protein molecule. Detection of antibodies against viral peptides bearing sequences identical to fragments of the human DNAH7 and CSMD2 proteins represents a necessary step in the validation of the molecular mimicry hypothesis.

To assess the immunodominance of two viral protein epitopes, an ELISA was performed using biotinylated 12-mer oligopeptide sequences encompassing the heptapeptides identified by bioinformatic analysis [57]. The predicted heptapeptides represent the minimal sequence required for B-cell receptor binding. However, in a natural context, this short fragment exists surrounded by neighboring amino acids. The use of 12-mer peptides allows the predicted heptapeptides to be placed in a more native context. Flanking sequences are important for proper spatial conformation and stability of antibody binding. To verify that the flanking residues did not introduce additional homologies with human proteins, the full 12-mer sequences were subjected to BLAST 2.17.0 analysis against the human proteome (UniProt). No significant additional homologies beyond those identified for the core heptapeptides were detected, confirming that the flanking residues did not confound the specificity of the assay. The assay utilized 12-mer oligopeptides (OPs) corresponding to E protein epitopes identified by bioinformatic analysis (HL, FE) and a control sequence corresponding to an HIV-1 Env protein epitope (GK). To minimize steric hindrance during antibody binding, an aminohexanoic acid spacer was used to link the peptide to biotin.

**Figure 1:**
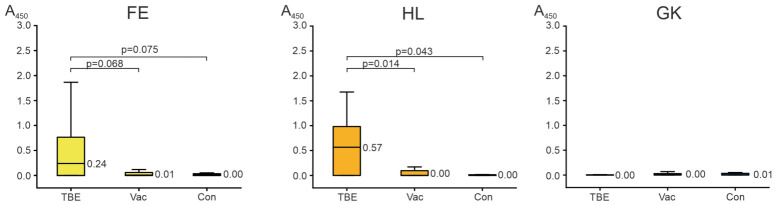
Immunogenicity of 12-mer peptide epitopes of the TBEV E protein (encompassing bioinformatically predicted heptapeptides) in three patient groups: TBE—patients with tick-borne encephalitis, Vac—vaccinated individuals, Con—control subjects with negative antibody titers against TBEV. Biotinylated 12-mer oligopeptides (HL, FE, GK) were used in the assay. The oligopeptide GK, which is not an E protein epitope, served as a control sequence.

Statistically significant differences in antibody concentrations against peptides HL and FE were observed between patient groups (*p* = 0.006). The results of pairwise comparisons are shown in Figure 1. Antibodies recognizing these oligopeptides were detected in patients with TBE. The binding of the tested oligopeptides to antibodies was found only in a subset of TBE patients. Among the 22 examined individuals, antibodies to peptide HL were detected in 11 (50.0%), and to peptide FE in 10 (45.5%) (optical density > 0.5). No specific antibodies to the control peptide GK were detected in any of the study groups.

The data obtained suggest that TBEV infection may lead to the generation of antibodies against epitopes possessing some similarity to human protein sequences. The production of such antibodies is an adverse event, as these antibodies, initially raised against the virus, may potentially recognize and cross-react with autoantigens, thereby triggering autoimmune processes in susceptible individuals [69]. However, our study also indicates that antibodies against the epitopes described herein are not generated in all individuals, but only in a subset of patients. This finding is consistent with studies demonstrating that the response to both infection and vaccination is characterized by high inter-individual variability and distinct patterns of immunodominance [70]. Furthermore, the presence of such antibodies does not necessarily imply the future development of an autoimmune disease, but rather indicates the presence of a risk.

Furthermore, no significant antibody levels against any of the tested peptides were detected in the plasma of individuals immunized with the Tick-E-Vac vaccine. The tick-borne encephalitis vaccine used in Russia (Tick-E-Vac) is based on the Sofjin strain [71] and contains a recombinant protein comprising domain III of the viral E protein [72]. Since the epitopes described herein are not part of this vaccine immunogen, the absence of detectable antibodies is expected (Figure 1). We emphasize that this observation is limited to the two peptides tested and does not constitute a comprehensive assessment of autoimmune safety.

Thus, this study demonstrates that natural TBEV infection, unlike vaccination with Tick-E-Vac, can potentially lead to the production of antibodies against two E protein epitopes. The existence of such antibodies creates a theoretical basis for the development of autoimmune responses via the mechanism of molecular mimicry.

The development of an autoimmune response may be associated not only with the generation of autoantibodies by B cells but also with the activation of T cells, which coordinate the immune response, including both the activation of B lymphocytes and the ability to destroy self-cells. Therefore, the potential role of T cells in the formation of an autoimmune response was analyzed next.

### 2.3. T-Cell-Dependent Antibody Response

B lymphocytes are capable of recognizing antigen epitopes via the B-cell receptor, leading to B-cell activation, clonal expansion, maturation, and subsequent antibody production, as well as antigen internalization, processing, and presentation to T cells in complex with MHC molecules. T cells scan MHC ligands presented on virtually all nucleated cells (expressing MHC class I molecules) and on professional antigen-presenting cells (expressing both MHC class I and II molecules). MHC–peptide complexes are generated through antigen processing and presentation pathways, which consist of a series of enzymatic events involving specialized organelles and processes that differ between MHC class I and MHC class II. MHC ligands (T-cell epitopes) trigger the T-cell immune response. T cells, particularly the CD4+ T helper cell population, can provide help to B cells in regulating antibody development and maturation. Upon epitope recognition, T cells proliferate, forming an effector population capable of recognizing the same epitope presented in the context of MHC molecules on other cells and generating long-lived memory populations that enable the host to respond rapidly to subsequent encounters with the same epitope [73]. Currently, various computational methods for T-cell epitope prediction have been developed.

The genes encoding MHC molecules (referred to as HLA in humans) are the most polymorphic in the human genome. Different MHC molecules exhibit distinct binding specificities, resulting in the presentation of different MHC ligands to T cells. In silico methods have been established for the identification and prediction of MHC-restricted T-cell epitopes. Neural networks utilize information on the amino acids that determine the MHC binding groove, as well as binding data for various MHC molecules. The NetMHCpan prediction method enables predictions to be made for MHC molecules that have never been experimentally tested [74].

To identify potential T-cell epitopes presented by HLA class II molecules, the IEDB resource was used [75,76]. The prediction algorithm generates 9-mer peptide sequences. From the library of 9-mer sequences, those containing the previously predicted heptapeptide motifs were selected. It was shown that the oligopeptide YEVGLEKLD can act as an epitope for HLA-DRB1*09:01, and VTGTQGTLL for HLA-DRB1*07:01 (Table 3). The use of 9-mer outputs from NetMHCIIpan is standard practice, as the algorithm identifies the 9-residue core that occupies the MHC II binding groove regardless of the input peptide length.

**Figure 2:**
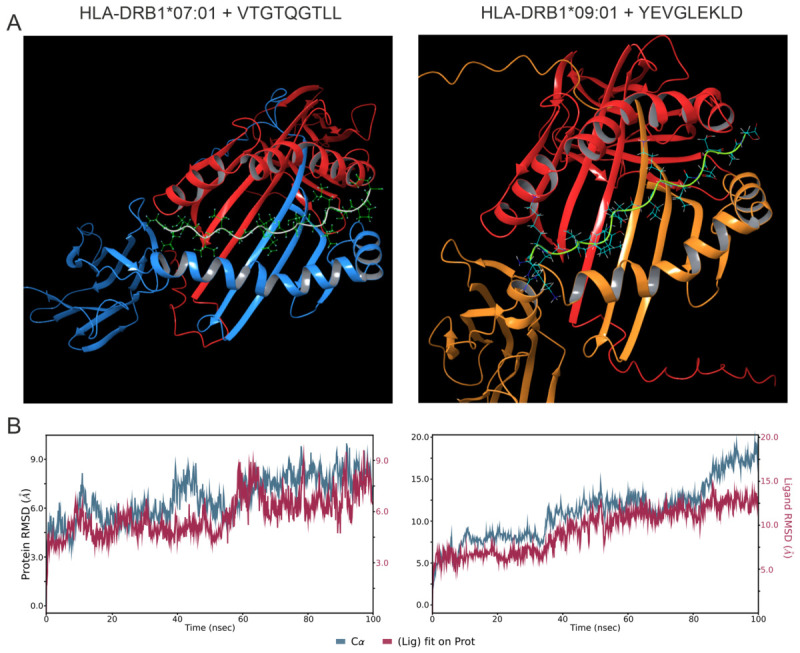
(**A**) Three-dimensional structures of HLA-II docked with the corresponding oligopeptide sequences. (**B**) RMSD plots of HLA–peptide complexes.

The three-dimensional structures of HLA molecules were generated using AlphaFold3 [78]. The HLA molecules were docked with the corresponding oligopeptide sequences. The stability of the resulting HLA II–peptide complexes was evaluated through 100 ns MD simulations, performed as previously described [79,80]. Complex stability and conformational dynamics were assessed using root mean square deviation (RMSD) analysis. Internal fluctuations of the peptides remained within 2 Å without major structural rearrangements, indicating conformational stability of the peptides within the binding groove and stable binding of these epitopes (Figure 2). The complexes exhibited stable charged, hydrophobic, and polar interactions, which, by duration, can be classified as highly persistent (>80% of simulation time), moderately persistent (50–80%), and less persistent (30–50%) (Table 4). The majority of key contacts were maintained for more than half of the trajectory, confirming the stability of the studied protein complex under molecular dynamics simulation conditions.

To evaluate the binding affinity of the predicted peptides to HLA molecules, the Gibbs free energy (ΔG) and dissociation constant (Kd) were calculated using the PRODIGY web server [81,82]. The results are summarized in Table 5. The binding energy values were −10.5 and −14.9 kcal/mol, indicating efficient interaction of all selected epitopes with their corresponding MHC class II alleles, with the binding of HLA-DRB1*09:01 to YEVGLEKLD being the strongest. The obtained ΔG values are in good agreement with previously reported data. For example, seven experimentally validated poxvirus epitopes binding to HLA-A*02:01 exhibited binding energies ranging from −9.3 to −12.9 kcal/mol [83].

Thus, HLA class II binding prediction demonstrated that the two oligopeptides YEVGLEKLD and VTGTQGTLL are capable of binding to HLA-DRB1*09:01 and HLA-DRB1*07:01, respectively. Theoretically, patients carrying either of these HLA alleles may be at risk of developing an autoimmune response.

## 3. Discussion

Molecular mimicry is considered one of the main mechanisms by which viral infections can disrupt immune tolerance and trigger autoimmune diseases [84]. This mechanism is based on the structural similarity between pathogen epitopes and host proteins [85]. Currently, over 7000 peer-reviewed articles have been published highlighting the contribution of molecular mimicry to autoimmune pathogenesis [86,87,88,89]. But the role of the TBEV in the development of autoimmunity has been studied very poorly. This study is aimed at elucidating the potential mechanisms of TBEV involvement in the development of autoimmunity through molecular mimicry.

A range of immunoinformatics approaches is currently available for protein analysis. One widely used strategy involves homology screening using BLAST to compare SCS derived from viral proteins against the human proteome. In the present study, the TBEV E protein amino acid sequence was fragmented into overlapping 7-mer peptides (shifting by one residue). This peptide library was then screened for matches within the human proteome, generating a list of peptides common to both TBEV E protein and human proteins. The obtained peptide list was then compared with the predicted B-cell epitopes for these proteins. This approach identified two viral epitopes (evglekl and vtgtqgt) potentially involved in eliciting a B-cell autoreactive response.

**Figure 3:**
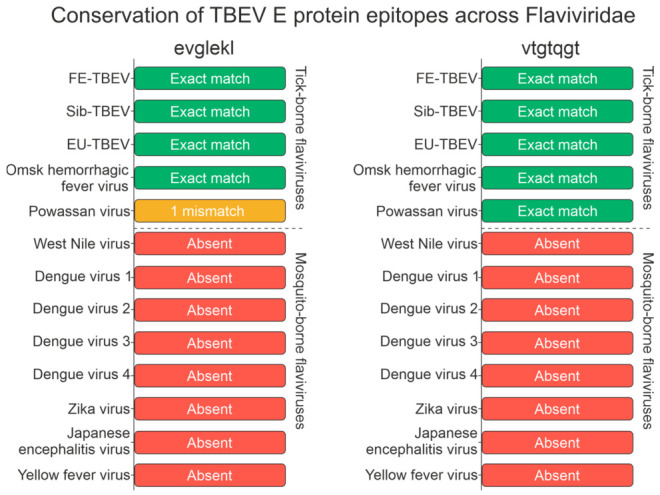
Conservation of TBEV E protein mimicry epitopes across the family Flaviviridae. Green: exact sequence identity (100%); orange: single amino acid substitution; red: epitope absent. A dashed line separates tick-borne (upper group) and mosquito-borne (lower group) flaviviruses.

To assess the specificity of the identified mimicry epitopes within the Flaviviridae family, we performed a conservation analysis of both heptapeptides (evglekl and vtgtqgt) across 13 representative flavivirus polyprotein sequences. Both epitopes were fully conserved across three TBEV subtypes (Far-Eastern, Siberian, and European) and the Omsk hemorrhagic fever virus. In the Powassan virus, vtgtqgt was fully conserved, while evglekl showed a single amino acid substitution (E1D; dvglekl). Notably, both epitopes were entirely absent from all mosquito-borne flaviviruses examined, including West Nile virus, Dengue virus serotypes 1–4, Zika virus, Japanese encephalitis virus, and Yellow fever virus (Figure 3). These results indicate that the identified mimicry epitopes are specific to tick-borne flaviviruses of the TBE serogroup, suggesting that the potential for autoimmune cross-reactivity via these sequences is restricted to tick-borne flavivirus infections.

Epitopes vary in their capacity to elicit antibody responses, with some exhibiting greater immunodominance than others [90]. To refine the antigenic landscape, we performed epitope mapping using plasma from patients with three clinical forms of TBE and from vaccine recipients. ELISA confirmed that the predicted epitopes are recognized by infection-induced antibodies, though only in a subset of individuals. By showing that the antibody response is directed precisely against linear viral peptides that share sequence identity with fragments of human DNAH7 and CSMD2, we fulfill the necessary first step in the validation of molecular mimicry. This peptide-based approach enables high-resolution mapping of immune recognition toward homologous epitopes, thereby establishing that TBEV infection can induce antibodies targeting viral sequences identical to those found in human proteins. Consequently, such antibodies have the potential to cross-react with autoantigens and may contribute to the initiation of autoimmune reactions.

DNAH7 encodes axonemal dynein heavy chain 7, a key component of respiratory cilia. Autoantibodies targeting ciliary components are associated with impaired mucociliary clearance and chronic airway inflammation [91]. The potential autoimmune phenotype linked to anti-DNAH7 antibodies may include chronic bronchitis and mucociliary dysfunction. CSMD2 is a synaptic transmembrane protein expressed predominantly in neurons. Autoantibodies against synaptic proteins are indeed key pathogenic factors in autoimmune encephalitis; they attack the neuronal cell surface, disrupting neuronal function [92]. Anti-CSMD2 antibodies could theoretically impair synaptic transmission, contributing to cognitive impairment, psychiatric symptoms, and encephalitic manifestations.

Assessing the autoimmune potential of viruses presents considerable challenges, as the manifestation of most autoimmune diseases may require 3 to 18 years [93]. An additional concern related to molecular mimicry arises from the incorporation of viral proteins or their epitopes into vaccine formulations. Several authors have raised concerns about vaccine-induced autoimmunity, providing examples where prior vaccinations triggered cross-reactive autoimmunity in susceptible populations [94,95]. Notably, during the swine flu outbreak in the United States in the late 1970s, influenza vaccination was associated with a markedly increased risk of Guillain–Barré syndrome via cross-reactivity [96]. Cross-reactivity between viral infections or vaccines and self-antigens has also been documented for hepatitis B virus and myelin proteins in multiple sclerosis, human papillomavirus and nuclear proteins in systemic lupus erythematosus, Coxsackie virus and islet cell proteins in type 1 diabetes, among others [97,98,99,100]. Consequently, Razim et al. [101] posited that cross-reactive epitopes should be eliminated during vaccine design to prevent potential future adverse outcomes.

The analysis of viral epitopes plays a critical role in vaccine design, and the inclusion of sequences homologous to human proteins in vaccine epitopes is discouraged owing to safety considerations. This study demonstrates that the Tick-E-Vac vaccine does not induce the same epitope-specific antibody responses observed during natural infection. Recognizing viruses as potential triggers highlights the importance of vaccination not only for preventing infection but also for mitigating its long-term sequelae, including autoimmune complications [102]. However, we emphasize that this observation is limited to the two peptides tested and does not constitute a comprehensive assessment of autoimmune safety.

Antibody production requires the activation of CD4+ helper T cells, which involves the interaction of the T-cell receptor (TCR) with the HLA class II complex on antigen-presenting cells [103]. B cells internalize and process antigens, load epitopes onto HLA class II molecules, and present the resulting HLA–peptide complexes to primed helper T cells. This interaction triggers T-cell cytokine release and drives B-cell differentiation into antibody-secreting plasma cells. Numerous associations between specific HLA allele carriage and susceptibility to autoimmune diseases have been documented. Most of these involve HLA class II, such as DRB1*15 in multiple sclerosis and DRB1*04 in rheumatoid arthritis [104]. In contrast, associations of HLA class I with the risk of autoimmune disorders are much rarer; the most characteristic is HLA-B*27 in ankylosing spondylitis, as well as in acute anterior uveitis [104,105,106].

Computational analysis suggests that HLA-DRB1*09:01 and DRB1*07:01 may theoretically facilitate the presentation of cross-reactive epitopes to CD4+ T cells in TBEV-infected individuals carrying these alleles. Furthermore, the interaction of HLA-DRB1*09:01 with its epitope exhibits greater strength compared to that of HLA-DRB1*07:01. These HLA molecules can bind TBEV peptides exhibiting similarity to the human proteome within their binding groove. Consequently, they may contribute to antigen presentation and immune activation.

HLA-DRB1*09:01 represents one of the most prevalent HLA-DRB1 alleles among Asian populations, whereas it occurs infrequently in Caucasian cohorts [107]. This allele has been implicated in the pathogenesis of various autoimmune conditions, including type 1 diabetes [108], juvenile myasthenia gravis [109], rheumatoid arthritis [110], and systemic lupus erythematosus [111]. Several studies indicate a higher risk of rheumatoid arthritis in individuals carrying the DRB1*09:01 allele [112]. Furthermore, HLA-DRB1*09 shows a strong association with anti-citrullinated protein antibody titers, and these associations were found to be independent of ethnic background [113].

While no association has been established between the HLA-DRB1*07:01 allele and autoimmune diseases, this allele is linked to hypersensitivity and allergic reactions. Notably, a higher incidence of hypersensitivity and the development of antibodies against asparaginase have been observed in patients harboring the HLA-DRB1*07:01 allele [114]. Furthermore, the DRB1*07 allele has been associated with psoriasis vulgaris [115].

These results highlight the importance of identifying disease-associated peptides presented by MHC/HLA molecules for a better understanding of autoimmune processes. Moreover, the results contribute to a better understanding of immunological and autoimmune processes that may arise as complications of viral infections.

While the in silico detection of homology between viral and human proteins is a prerequisite, it is not sufficient for the development of autoimmune pathology. The manifestation of such a scenario depends on the convergence of multiple factors: a critical degree of structural epitope similarity, genetic susceptibility (particular MHC alleles), and environmental triggers capable of breaching peripheral immune tolerance mechanisms.

### Limitations

We acknowledge that demonstrating cross-reactivity of patient antibodies with native human DNAH7 and CSMD2 proteins would constitute direct evidence of molecular mimicry. However, obtaining full-length recombinant proteins is fraught with technical difficulties. This was beyond the technical and resource capabilities of this study.

A limitation of the present study is the absence of HLA genotyping data for the patient cohort. Since only plasma samples were available, DNA-based HLA typing could not be performed. Consequently, the association between HLA-DRB1*09:01 and DRB1*07:01 alleles and antibody responses in TBE patients remains theoretical and is based solely on in silico predictions. The statement that carriers of these alleles ‘may be at risk’ should be interpreted strictly as a computational hypothesis.

In our study, plasma samples were collected exclusively during the acute phase of tick-borne encephalitis, whereas the onset of autoimmune complications often occurs several years later. This time lag is a major obstacle to direct analysis of the delayed autoimmune outcomes of viral infection. Therefore, in this study, by “risk factor” we mean not the diagnosis of established autoimmune disease, but the detection of antibodies to viral peptides identical to fragments of human autoantigens, which may predispose to a potential breakdown of tolerance. We consider this a necessary but not sufficient condition for the initiation of an autoimmune process.

Although the sample size (22 patients with tick-borne encephalitis) limits the statistical power of the study, the study was exploratory in nature and aimed to identify candidate epitopes of molecular mimicry and assess their immunodominance. The obtained results provide a basis for future, larger-scale studies.

## 4. Materials and Methods

### 4.1. TBEV E Protein Sequence Acquisition and B-Cell Epitope Prediction by ABCpred

TBEV E protein sequences were obtained from the GenBank database (https://www.ncbi.nlm.nih.gov/, accessed on 7 May 2025). Since the Siberian and Far-Eastern subtypes circulate in the Siberian region of Russia [116], the following virus variants were used in this study:

Siberian subtype (GenBank: AEQ77277.1 [117]);

Far-Eastern subtype (GenBank: AFV41132.1);

Far-Eastern subtype (GenBank: AEP25267.2 [71]).

Sequence comparisons among the E proteins of different subtypes were conducted via multiple sequence alignment using Clustal Omega 1.2.4, accessible through the EMBL-EBI portal (https://www.ebi.ac.uk/, accessed on 7 May 2025).

The homology between TBEV E proteins and human proteins was assessed using the short contiguous sequence (SCS) approach, as described in our previous study [79]. Briefly, each of the three E protein sequences was fragmented into overlapping heptapeptides with a one-residue shift (e.g., srcthle, rcthlen, cthlenr, thlenrd) using Python 3.13. A unified library of unique 7-mers was then created by removing duplicates. This library was then screened against the human proteome for homologous sequences using the UniProtKB peptide search tool (https://www.uniprot.org/peptide-search, accessed on 7 May 2025) [118,119].

B-cell epitopes were predicted using the ABCpred server (https://webs.iiitd.edu.in/raghava/abcpred/, accessed on 12 May 2025). B-cell epitopes with a Score > 0.7 were selected.

### 4.2. Donors and Patients

Blood plasma samples were obtained from TBE patients hospitalized at the City Infectious Diseases Clinical Hospital No. 1 (Novosibirsk). Samples from vaccinated subjects and control donors were collected at the ICBFM SB RAS. Control plasma was derived from donors with no history of TBE infection or vaccination. The study protocol was approved by the Local Ethics Committee of the Institute of Chemical Biology and Fundamental Medicine (Protocol Number 26-7 from 24 April 2025). All participants (patients and healthy donors) were informed of the study’s purpose and provided written consent for blood donation, in compliance with the Helsinki Declaration.

Blood samples were centrifuged at 3000× *g* for 10 min. The separated plasma was frozen and stored until antibody analysis. Anti-TBEV antibody titers were measured using the D-1156 assay kit (Vector-Best, Novosibirsk, Russia), following the manufacturer’s protocol.

### 4.3. Assessment of the Immunodominance of Individual Epitopes

Four biotinylated 12-mer oligopeptides were synthesized by SmartSynthetics LLC (St. Petersburg, Russia); their sequences are listed in Table 6. Each peptide was C-terminally labeled with biotin via an aminohexanoic acid spacer.

Ninety-six-well high-binding plates were coated overnight at 4 °C with avidin (2 μg/mL in carbonate buffer). After blocking with 1% non-fat milk/PBS-T (60 min, 37 °C) and washing, biotinylated peptides (40 ng/mL) were added for 30 min. Washed plates were incubated with 1:10 diluted plasma (30 min, shaking), then with HRP-conjugated anti-human IgG (1:10,000, 30 min, shaking). Following washes, TMB substrate was added; the reaction was stopped with 1 M H_2_SO_4_. OD was measured at 450/620 nm (Multiskan FC, Thermo Scientific, Waltham, MA, USA). Results are means of three independent experiments. A sample was classified as seropositive if its OD value exceeded the mean OD of the control group plus two standard deviations (mean + 2SD).

The Shapiro–Wilk test was used to assess the distribution of the data. Since the distribution deviated from normality, comparisons among the three groups were performed using the nonparametric Kruskal–Wallis test, followed by Dunn’s post hoc test for pairwise comparisons.

### 4.4. Prediction of HTL (Helper T Lymphocyte) Epitopes

MHC-II-binding epitopes were predicted using the NetMHCpan algorithm [77] on the IEDB platform [75,76] (http://tools.iedb.org/mhcii/, accessed on 24 November 2025). The algorithm generates 9-mer core binding sequences from the input peptide. The following allele–peptide pairs were identified as strong binders (percentile rank < 2%). The obtained peptides were then screened for heptapeptide motifs.

### 4.5. Molecular Docking and Molecular Dynamics

Molecular dynamics (MD) simulations followed established protocols [80,120] and comprised several stages. Initially, the protein complex structure was imported in PDB format and prepared using the ‘Protein Preparation Workflow’. Chains were separated for subsequent protein-protein docking with the corresponding module, where chains A and B served as the receptor and chain C as the ligand. Key docking parameters included 70,000 ligand rotations for conformational sampling and a maximum of 30 returned poses. Poses with the peptide correctly positioned within the HLA binding groove were selected visually.

Protein preparation and minimization employed default parameters within the ‘Protein Preparation Workflow’. Hydrogen bond optimization and final restrained minimization were conducted at pH 7.4 using the OPLS4 force field [121] with full hydrogen atom optimization. The RMSD limit for heavy atoms from initial coordinates was 0.30 Å.

For MD simulations, the selected HLA–peptide complex was placed in an orthorhombic box with a 15 Å buffer distance from the protein surface. Solvation utilized the TIP3P water model, and NaCl was added to achieve 0.15 M physiological ionic strength, with additional Na^+^ and Cl^−^ ions for charge neutralization. MD simulations were conducted with Desmond 7.2 for 100 ns using a 2 fs integration time step at 310 K. The NPT ensemble was maintained with the Martyna–Tobias–Klein barostat and Nosé–Hoover thermostat. A total of 1000 trajectory frames were saved for subsequent analysis. All calculations were performed on the SRF SKIF computing cluster.

### 4.6. Binding Affinity Analysis

Binding affinities, expressed as dissociation constants (Kd) and corresponding Gibbs free energy changes (ΔG), were calculated for the molecular complexes at 37 °C using the PRODIGY web server [81,82] (https://rascar.science.uu.nl/prodigy/, accessed on 28 January 2026).

## 5. Conclusions

The field of immunoinformatics is rapidly advancing, offering tools that can replace or supplement expensive and labor-intensive screening assays, thereby substantially accelerating research [122]. In this study, we combined bioinformatic predictions with a targeted experimental approach (ELISA employing two biotinylated peptides). This integrated in silico and in vitro strategy therefore provides a feasible framework for investigating molecular mimicry and identifying autoreactive epitopes potentially implicated in virus-associated autoimmune disorders.

In this study, the autoimmune potential of the TBEV E protein was assessed. Two viral epitopes (evglekl and vtgtqgt) were identified within the E protein. These epitopes exhibit sequence identity with fragments of the human proteins DNAH7 and CSMD2. ELISA experiments confirmed the immunogenicity of these epitopes during natural TBEV infection, with antibodies observed in a proportion of TBE patients, but not in all. Importantly, these antibodies were absent in recipients of the Tick-E-Vac vaccine, suggesting that the current vaccine formulation does not elicit cross-reactive humoral responses against these particular epitopes.

In addition, computational analysis indicated that these epitopes may be presented by HLA class II molecules (DRB1*09:01 and DRB1*07:01). Molecular dynamics simulations confirmed stable binding of the corresponding peptides within the HLA binding grooves, with favorable binding energies. These findings point to a potential role for T-helper cells in mediating the autoreactive response.

In comparison with SARS-CoV-2, the TBEV E protein displays substantially fewer homologous sequences to the human proteome, suggesting a comparatively lower autoimmune potential. Nevertheless, the existence of immunodominant cross-reactive epitopes in a subset of infected individuals provides a theoretical foundation for autoimmune reactions through molecular mimicry.

The in silico prediction of homologous regions is not direct evidence of their pathogenic potential. The triggering of an autoimmune response through molecular mimicry is a complex, multifactorial phenomenon. Its realization requires a combination of several factors, including a critical level of structural similarity between epitopes, genetic predisposition, and triggering environmental influences capable of overcoming the mechanisms of peripheral immune tolerance.

Thus, natural TBEV infection may elicit antibodies against epitopes sharing homology with human proteins, especially in genetically susceptible hosts. While the identification of such homologous regions does not predict the onset of autoimmune disease, it constitutes a risk factor that merits further investigation. These findings highlight the significance of vaccination, not only in preventing acute TBEV infection but also in reducing the risk of potential long-term autoimmune consequences.

## Figures and Tables

**Table 1:** Percent Identity Matrix for E proteins of different TBEV subtypes, created using Clustal2.1.

E Protein of Different TBEV Subtypes	FE-TBEV (GenBank: AEP25267.2	Sib-TBEV (GenBank: AEQ77277.1	FE-TBEV (GenBank: AFV41132.1)
FE-TBEV (GenBank: AEP25267.2	100.00	96.57	99.20
Sib-TBEV (GenBank: AEQ77277.1	96.57	100.00	97.38
FE-TBEV (GenBank: AFV41132.1)	99.20	97.38	100.00

**Table 2:** Occurrence of TBEV E protein heptapeptides in the human proteome.

SCS	UniProtKB ID	Match	Gene Names	Occurrence Within the Epitopes of the E Protein	Occurrence Within the Epitopes of Human Proteins
evglekl	Q8WXX0	Positions 2589–2595: evglekl	*DNAH7*	YES	YES
gflpkll	Q9BZZ2	Positions 2–8: gflpkll	*SIGLEC1*	Only Sib-TBEV	NO
gpatlae	A0A590UJF1	Positions 255–261: gpatlae	*STRA8*	YES	YES
ktlehlp	Q9BYW2	Positions 1802–1808: ktlehlp	*SETD2*	Only Sib-TBEV	YES
lagglvl	Q93050	Positions 769–775: lagglvl	*VPP1*	NO	NO
sceakkk	O75949	Positions 365–371: sceakkk	*NALF2*	NO	YES
thsgrkt	Q9NZV7	Positions 377–383: thsgrkt	*ZIM2*	YES	YES
vlelggc	Q8N1E6	Positions 147–153: vlelggc	*FBXL14*	YES	NO
vtgtqgt	Q7Z408	Positions 1102–1108: vtgtqgt	*CSMD2*	YES	YES

**Table 3:** Inclusion of homologous heptapeptides in the sequence of HTL epitopes of 6 human proteins, with the corresponding HLA class II alleles, predicted binding scores, and percentile ranks calculated using the NetMHCpan algorithm [77] on the IEDB platform.

Allele	Core Peptide	Score	Rank
HLA-DRB1*09:01	YEVGLEKLD	0.6056	1.80
HLA-DRB1*07:01	VTGTQGTLL	0.7304	0.87

**Table 4:** Summary of interactions observed during molecular dynamics simulations.

MHC Class II Molecule Presenting an HTL Epitope	Charged *, **	Hydrophobic	Polar	Additionally
HLA-DRB1*07:01	B: GLU 42 (50%) A: ARG 85 (49%) B: ARG 85 (37%) A: ARG 85 (33%)	B: TRP 75 (96%) B: TYR 27 (85%) A: TRP 75 (42%) A: TYR 27 (33%) B: VAL 71 (32%)	B: ASN 96 (98%) A: ASN 96 (98%) A: ASN 96 (80%) B: GLN 88 (63%) A: GLN 88 (41%) A: GLN 88 (37%) B: GLN 78 (30%)	
HLA-DRB1*09:01	B: ARG 85 (93%) A: ASP 25 (79%) B: ASP 25 (75%) A: ARG 85 (67%) B: ARG 85 (61%) A: ASP 25 (58%) B: GLU 88 (55%) B: GLU 101 (48%) B: GLU 88 (46%) B: ASP 25 (45%) B: GLU 88 (42%) B: ARG 84 (40%) A: GLU 101 (39%) B: ARG 84 (39%) B: GLU 101 (37%) B: GLU 88 (32%) A: LYS 23 (31%) B: GLU 88 (30%)	B: TRP 75 (61%) B: TRP 75 (55%)	B: ASN 96 (48%) A: ASN 96 (47%) A: ASN 96 (41%) A: ASN 96 (30%)	B: GLY 100 (46%)

* Interactions occurring for more than 30.0% of the simulation time throughout the selected trajectory (from 0.00 to 100.00 ns) are shown. ** It is possible to have interactions with >100% as some residues may have multiple interactions of a single type with the same ligand atom.

**Table 5:** Gibbs free energy (ΔG) and dissociation constant (Kd) between docked oligopeptides and HLA class II, calculated using the PRODIGY web server.

MHC Class II Molecule Presenting an HTL Epitope	ΔG (kcal mol^−1^)	Kd (M) at 37 °C
HLA-DRB1*07:01 + VTGTQGTLL	−10.5	4.20 × 10^−8^
HLA-DRB1*09:01 + YEVGLEKLD	−14.9	2.90 × 10^−1^

**Table 6:** Heptapeptides and corresponding E protein epitopes.

Protein	Abbreviation	Heptapeptides	12-mer Oligopeptides Coated onto the Plate Wells
DNAH7	HL	evglekl	Biotin-Ahx-HVTCEVGLEKLKMKGL
CSMD2	FE	vtgtqgt	Biotin-Ahx-FVTGTQGTTRVTLVLE
Control peptide sequence	GK	-	Biotin-Ahx-GGDMRDNWRSELYK

## Data Availability

Data is contained within the article.

## Abbreviations

Kd	dissociation constant
EU-TBEV	European tick-borne encephalitis virus
FE-TBEV	Far Eastern tick-borne encephalitis virus
HTL	helper T lymphocyte
MD	molecular dynamics
OPs	oligopeptides
OD	optical density
RMSD	root mean square deviation
SCS	short contiguous sequence
Sib-TBEV	Siberian tick-borne encephalitis virus
TCR	T-cell receptor
TBE	tick-borne encephalitis
TBEV	tick-borne encephalitis virus
